# uPA‐derived peptide, Å6 is involved in the suppression of lipopolysaccaride‐promoted inflammatory osteoclastogenesis and the resultant bone loss

**DOI:** 10.1002/iid3.169

**Published:** 2017-05-11

**Authors:** Yosuke Kanno, Chihiro Maruyama, Ayaka Matsuda, Akira Ishisaki

**Affiliations:** ^1^ Faculty of Pharmaceutical Science Department of Clinical Pathological Biochemistry Doshisha Women's Collage of Liberal Arts Kyoto Japan; ^2^ Department of Biochemistry Iwate Medical University School of Dentistry Morioka Iwate Japan

**Keywords:** Bone loss, osteoclasts, uPA‐derived peptide Å6

## Abstract

**Introduction:**

Chronic inflammatory diseases such as rheumatoid arthritis and periodontitis frequently cause bone destruction. Inflammation‐induced bone loss results from the increase of bone‐resorbing osteoclasts. Recently, we demonstrated that urokinase type plasminogen activator (uPA) suppressed lipopolysaccaride (LPS)‐inflammatory osteoclastogenesis through the adenosine monophosphate‐activated protein kinase (AMPK) pathway, whereas its receptor (uPAR) promoted that through the Akt pathway.

**Methods:**

We investigated the effects of uPA‐derived peptide (Å6) in the LPS‐induced inflammatory osteoclastogenesis and bone destruction.

**Results:**

We found that Å6 attenuated inflammatory osteoclastogenesis and bone loss induced by LPS in mice. We also showed that Å6 attenuated the LPS‐promoted inflammatory osteoclastogenesis by inactivation of NF‐κB in RAW264.7 mouse monocyte/macrophage lineage cells. Furthermore, we showed that Å6 attenuated the Akt phosphorylation, and promoted the AMPK phosphorylation.

**Conclusion:**

Å6 is involved in the suppression of LPS‐promoted inflammatory osteoclastgensis and bone destruction by regulating the AMPK and Akt pathways. These findings provide a basis for clinical strategies to improve the bone loss caused by inflammatory diseases.

## Introduction 

Urokinase‐type plasminogen activator (uPA) and its receptor (uPAR) have the proteolytic function which converts plasminogen (Plg) to plasmin 1, and then plasmin not only degrade fibrin and any ECM proteins but also activate matrix metalloproteinases and growth factors 2. uPA and uPAR also promotes the intracellular signaling by the interaction with transmembrane proteins such as integrins, and mediate cellular adhesion, differentiation, proliferation, and migration 3. These functions of uPA and uPAR are associated with the development of inflammatory diseases such as rheumatoid arthritis, periodontitis, cancer, and fibrosis 4, 5, 6, 7, 8, 9, 10. Recently, we demonstrated that uPA suppressed inflammatory osteoclastogenesis through the adenosine monophosphate‐activated protein kinase (AMPK) pathway 11. Conversely, uPAR promoted inflammatory osteoclastogenesis through the Akt pathway, and the blocking of uPAR attenuated them 12.

Å6 is an 8‐mer capped peptide derived from uPA (amino acid 136–143, KPSSPPEE), and inhibits the interaction of uPA with uPAR 13. It has been reported that Å6 inhibits tumor growth, tumor metastasis and angiogenesis 13, 14, 15. Å6 also inhibits hypoxia‐induced retinal neovascularization and choroidal neovascularization 16, 17. Several clinical studies have been shown that Å6 was well tolerated, and no toxicity in Phase 1 and 2 clinical trials 18, 19, 20, 21. Although the molecular mechanism of Å6 remains to be clarified, the inhibition of uPA and uPAR interaction by Å6 may affect the amelioration of various diseases.

We herein reported the effects of uPA‐derived peptide, Å6 on the lipopolysaccaride (LPS)‐induced inflammatory OC formation and the resultant bone loss.

## Material and Methods

The animal experiments in this study were approved by the Animal Research Committee of Doshisha Women's Collage of Liberal Arts (Approval ID: Y15‐025). All experiments were performed in accordance with relevant guidelines and regulations.

### Reagents

LPS (from *Escherichia coli* 0111:B4) was purchased from Sigma–Aldrich (St. Louis, MO). Å6 peptide (KPSSPPEE) was synthesized by GL Biochem (Shanghai, China).

### Animals

C57B6J mice littermates were housed in groups of two to five in filter‐top cages with a fixed 12 h light, 12 h dark cycle.

### Bone destruction by the administration of LPS in mice

LPS (25 mg/kg) and Å6 (50 mg/kg) were administered subcutaneously into the shaved back of the male mice. The administration was carried out weekly for up to 4 weeks.

### Bone histology

Bone histomorphometry of femurs in male mice were performed as previously described 12. Each femur was removed and fixed in 4% paraformaldehyde for 2 days, and then demineralized with 10% EDTA for 14 days before embedding in paraffin. Paraffin‐embedded tissue was serially sectioned at 4–7 μm distances. Then, the sections were stained with TRAP by using TRAP kit (Sigma–Aldrich).

For the quantitative evaluation of the intensity of TRAP‐staining in decalcified sections of femurs from the mice, the TRAP‐stained images obtained from separate fields on the specimens were analyzed by using ImageJ 1.43u.

### Measurement of bone mineral density

Bone mineral density (BMD) was measured as previously described 11, 22. The BMD of femurs from mice at the indicated time was evaluated by using peripheral quantitative computed tomography with a fixed X‐ray fan beam of 50‐μm spot size, at 1 mA and 50 kVp (LaTheta LCT‐100S; Aloka, Tokyo, Japan).

### Cell culture and OC differentiation

RAW264.7 mouse monocyte/macrophage lineage cells were maintained in α‐MEM supplemented with 10% fetal calf serum (FCS) and 1% penicillin‐streptomycin at 37°C in a humidified atmosphere of 5% CO_2_/95% air. OC formation was induced as previously described 11, 12. RAW 264.7 cells were cultured for 3 days with LPS (1 μg/ml) and M‐CSF (100 ng/ml) in the absence or presence of Å6 (100 μM) in 48‐well plates.

### siRNAs study

RAW 264.7 cells were transfected with uPA or uPAR siRNA (Santa Cruz Biotechnology, Santa Cruz, CA) using Lipofectamine 2000 (Invitrogen, Carlsbad, CA) according to the manufacturer's instructions. A nonspecific siRNA (Santa Cruz Biotechnology) was employed as the control.

### Western blot analysis

Western blot analysis was performed as previously described 23. Cells were washed twice with cold PBS, harvested, and then sonicated in lysis buffer containing 10 mM Tris–HCl buffer (pH 7.5), 1% SDS, 1% Triton X‐100, and a protease inhibitor cocktail (Roche, Mannheim, Germany). The protein concentration in each lysate was measured using a BCA protein assay kit (Pierce, Rockford, IL). Proteins in the supernatant were separated by electrophoresis on 10% SDS‐polyacrylamide gels and transferred to a PVDF membrane. We detected expressions of TRAP, NFATc1, IκBα, uPA, uPAR, GAPDH, phospho‐AMPK, AMPK, phospho‐Akt, Akt by using anti‐TRAP antibody (Santa Cruz Biotechnology, Dallas, TX), anti‐NFATc1 antibody (Santa Cruz Biotechnology), anti‐IκBα antibody (IMGENEX, San Diego, CA), anti‐uPA antibody (Santa Cruz Biotechnology), anti‐uPAR antibody (Santa Cruz Biotechnology), anti‐GAPDH antibody (Sigma–Aldrich), anti‐phospho‐AMPK antibody (Cell Signaling Technology, Danvers, MA), anti‐AMPK antibody (Cell Signaling Technology), anti‐phospho‐Akt antibody (Cell Signaling Technology), anti‐Akt antibody (Cell Signaling Technology) followed incubation with horseradish peroxidase‐conjugated antibody to rabbit IgG (Amersham Pharmacia Biotech, Uppsala, Sweden).

### Dual luciferase reporter assay

Dual luciferase reporter assay was performed as previously described 11. pGL4.32 (luc2P/NF‐κB/Hygro) vector contains five copies of NF‐κB response element (NF‐κB‐RE) that derives transcription of the luciferase reporter gene luc2P (Promega, Madison, WI, USA). RAW264.7 cells were co‐transfected with pGL4.32 (luc2P/NF‐κB/Hygro) vector and the internal control vector pGL4.74 (hRluc/TK) using the Lipofectamine 2000 transfection reagent (Invitrogen) according to the manufacturer's protocol. At 24 h post‐transfection, the cells were stimulated with described reagents, and then assayed for luciferase activity using the Dual‐Glo luciferase assay system (Promega) according to the manufacturer's protocol.

### ELISA

RAW 264.7 cells were cultured for 24 h with LPS (1 μg/ml). After the indicated incubation periods, the conditioned medium was collected, and the TNF‐α in the medium was then measured using a TNF‐α mouse antibody pair (Invitrogen). The absorbance of the ELISA samples was measured at 450 nm using Multiskan JX (Thermo Labsystems, Waltham, MA).

### Statistical analysis

All data are expressed as mean ± SEM. The significance of the effects of each treatment (*p* < 0.05) was determined by analysis of variance 24 followed by the least significant difference test.

## Results

### Å6 attenuated inflammatory osteoclastogenesis and bone destruction induced by LPS in mice

**Figure 1:**
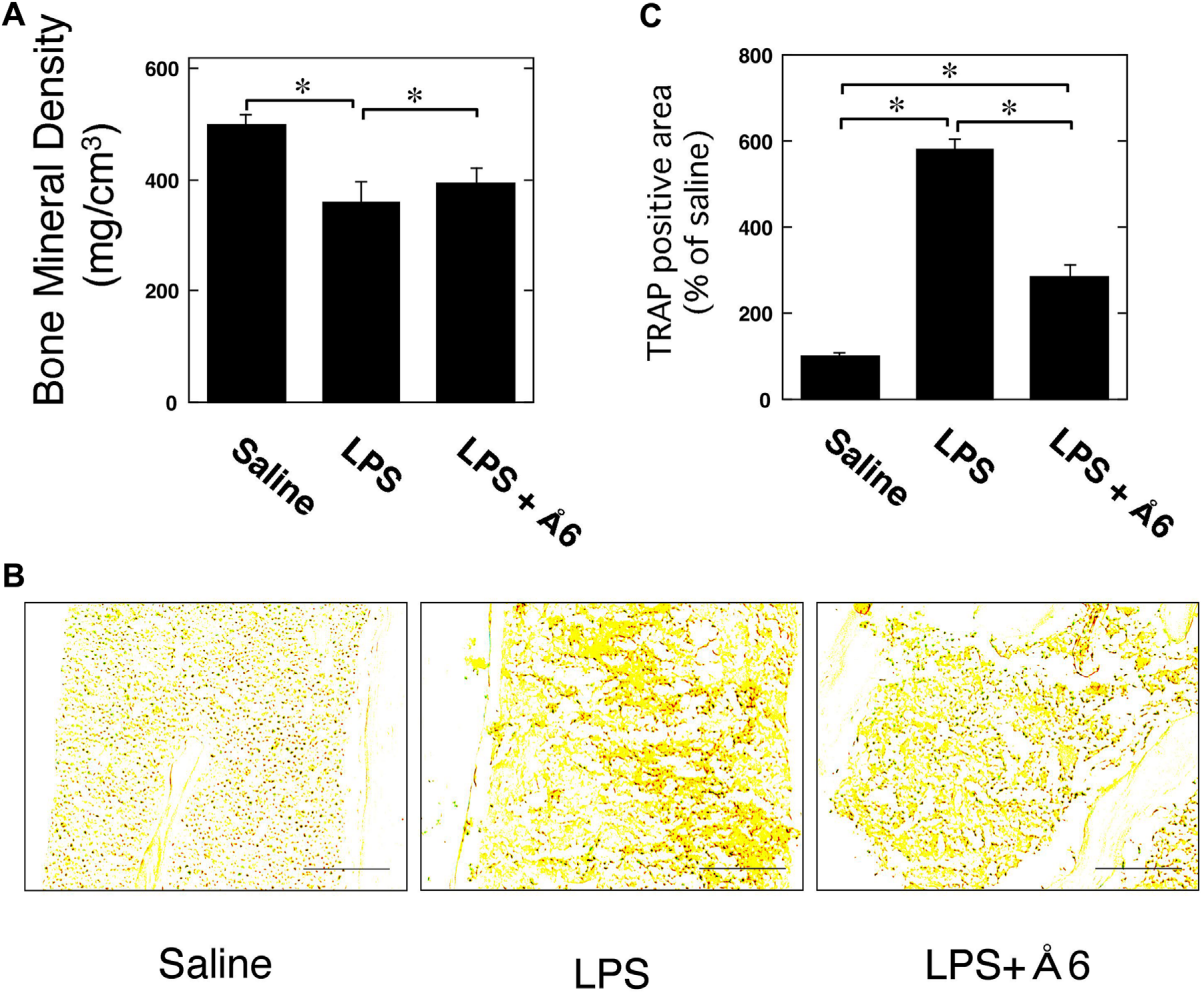
Å6 attenuated inflammatory osteoclastogenesis and bone destruction induced by LPS in mice. (A) 25 mg/kg LPS alone or 25 mg/kg LPS and 50 mg/kg Å6 was administered subcutaneously into the shaved back of the male C57B6J mice. Administration in the same site was carried out weekly for up to 4 weeks. Trabecular BMD in the femur of male wild‐type mice was obtained from pQCT measurement (*n* = 18). (B) The TRAP‐staining in the femur of male wild‐type mice. Scale bar = 100 μM. (C) The intensity of TRAP‐staining in the femur of male C57B6J mice was quantitatively evaluated as described in the Materials and Methods (*n* = 4). The data represent the mean ± SEM. *p < 0.01.

To clarify the effects of Å6 in the inflammatory osteoclastogenesis and bone destruction, we examined the bone mineral density (BMD) in the mice by the administration of lippolysaccaride (LPS), which is a well‐known pathogen of inflammatory bone loss 25. Å6 attenuated the decrease of BMD induced by LPS (Fig. 1A). Additionally, Å6 attenuated the increase of TRAP‐positive area induced by LPS (Fig. 1B and C).

### Å6 attenuated OC differentiation of macrophage RAW264.7 cells promoted by LPS

**Figure 2:**
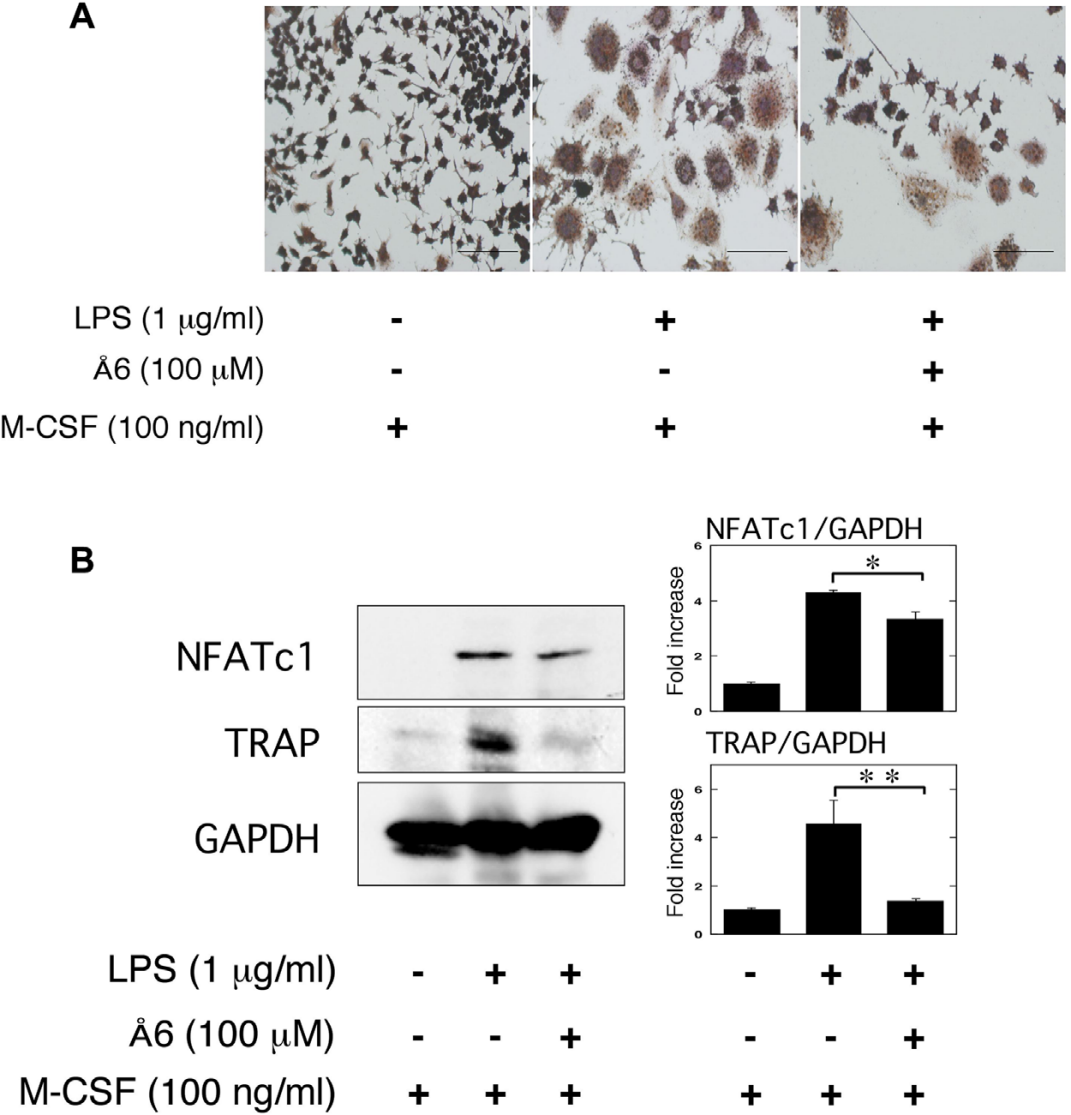
Å6 attenuated OC differentiation of macrophage RAW264.7 cells promoted by LPS. RAW264.7 cells were cultured for 3 days in the absence or presence of LPS (1 μg/ml), M‐CSF (100 ng/ml), or Å6 (100 μM) as indicated. (A) TRAP‐staining was performed to detect OC differentiation. Scale bar = 100 μm. (B) The expression of NFATc1 and TRAP in RAW264.7 cells was examined by a Western blot analysis. The histogram on the right panel shows quantitative representations of NFATc1 or TRAP obtained from densitometry analysis after normalization to the levels of GAPDH expression (*n* = 3). The data represent the mean ± SEM. *p < 0.01, **p < 0.05.

We also examined that the effects of Å6 in LPS‐induced the OC differentiation of RAW264.7 cells. RAW264.7 cells simultaneously treated with LPS and M‐CSF (Fig. 2A, center panel) looked more clearly positive against TRAP staining than the cells treated with M‐CSF alone (Fig. 2A, left panel), but did not appear to be typically and predominantly enlarged multi‐nuclear cells containing more than three nuclei in each cell like as maturely differentiated OCs stimulated with LPS and M‐CSF. In addition, the cell number of TRAP‐positive and enlarged RAW264.7 cells under the treatment with LPS and M‐CSF was moderately decreased by administration of Å6 (Fig. 2A, right panel). Additionally, the stimulation with LPS and M‐CSF more clearly induced the expression of OC markers, NFATc1 and TRAP than stimulation with M‐CSF alone (Fig. 2B). In addition, the LPS‐promoted OC differentiation of RAW264.7 cells under the M‐CSF treatment was clearly suppressed by administration of Å6. These data suggest that Å6 treatment attenuated the LPS‐promoted OC differentiation of macrophage under the M‐CSF treatment.

### Å6 attenuated TNF‐α secretion induced by LPS from macrophage RAW264.7 cells

**Figure 3:**
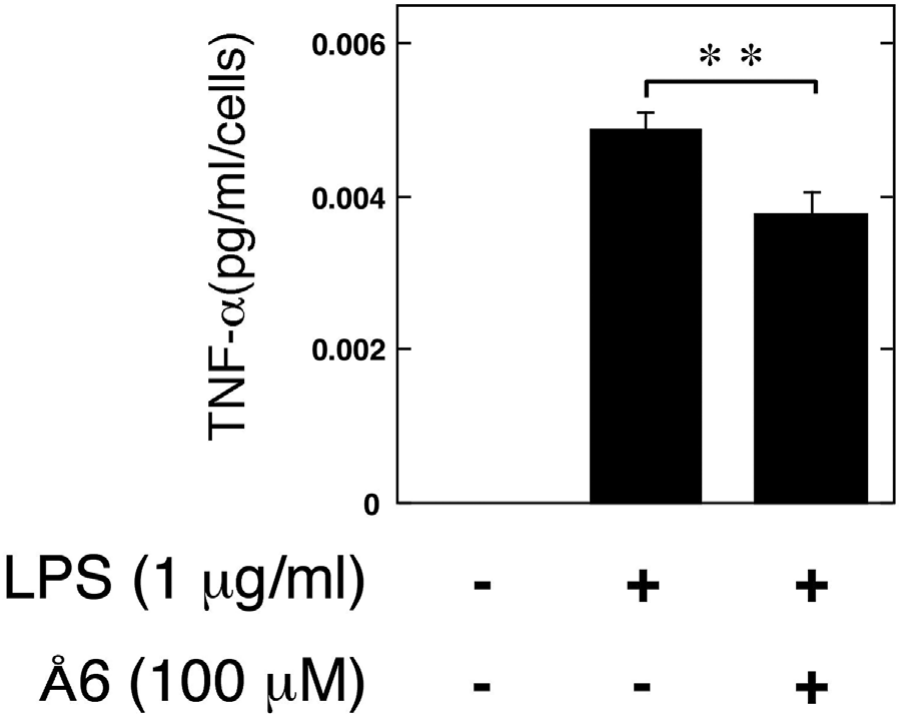
Å6 attenuated TNF‐α secretion induced by LPS from macrophage RAW264.7 cells. RAW264.7 cells were cultured for 24 h in the absence or presence of LPS (1 μg/ml) or Å6 (100 μM) as indicated. The TNF‐α content in the conditioned media of RAW264.7 cells was determined by using ELISA as described in Materials and Methods (*n* = 3). The data represent the mean ± SEM. **p < 0.05

It has been reported that LPS‐stimulated osteoclastogenesis is mediated by TNF‐α 26, 27. To clarify the effects of Å6 in the LPS‐induced TNF‐α production, we examined that the TNF‐α production in RAW264.7 cells. Å6 attenuated the LPS‐induced TNF‐α secretion from RAW264.7 cells (Fig. 3).

### Å6 attenuated NF‐κB activation induced by LPS in macrophage RAW264.7 cells

**Figure 4:**
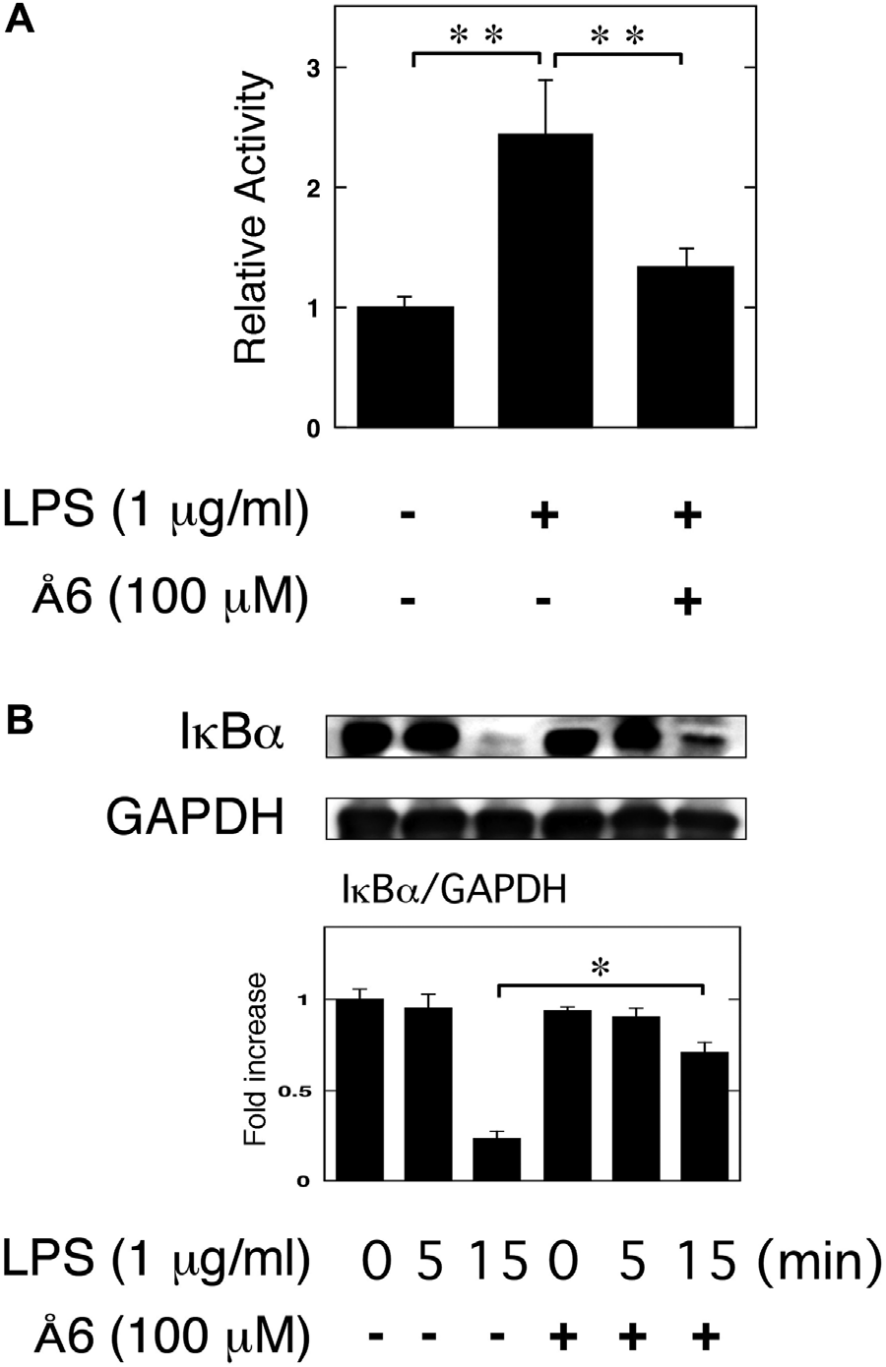
Å6 attenuated NF‐κB activation induced by LPS in macrophage RAW264.7 cells. (A) RAW264.7 cells were co‐transfected with a Fluc reporter plasmind containing NF‐κB promoter and the internal control vector pRL‐TK. At 24 h after the transfections, these cells were cultured in the presence or absence of 100 μM Å6 for 30 min, and then stimulated with 1 μg/ml LPS for 3 h. Finally, the transcriptional activity of NF‐κB was performed (*n* = 3). (B) RAW264.7 cells were pretreated with 100 μM Å6 for 30 min and then stimulated with 1 μg/ml LPS for the indicated periods. Degradation of IκBα was evaluated by a Western blot analysis using anti‐ IκBα antibody. The histogram on the bottom panel shows quantitative representations of IκBα obtained from densitometry analysis after normalization to the levels of GAPDH expression (*n* = 3). The data represent the mean ± SEM. *p < 0.01, **p < 0.05.

We examined the effects of Å6 on the LPS‐induced NF‐κB transcriptional activity through the use of a functional promoter assay with NF‐κB‐responsive element. Å6 attenuated the LPS‐induced NF‐κB activation (Fig. 4A). We also confirmed that Å6 attenuated the LPS‐induced IκBα degradation (Fig. 4B). These data strongly suggest that Å6 inhibited the LPS‐activated NF‐κB signaling.

### Å6 inhibited the Akt pathway, but activated the AMPK pathway

**Figure 5:**
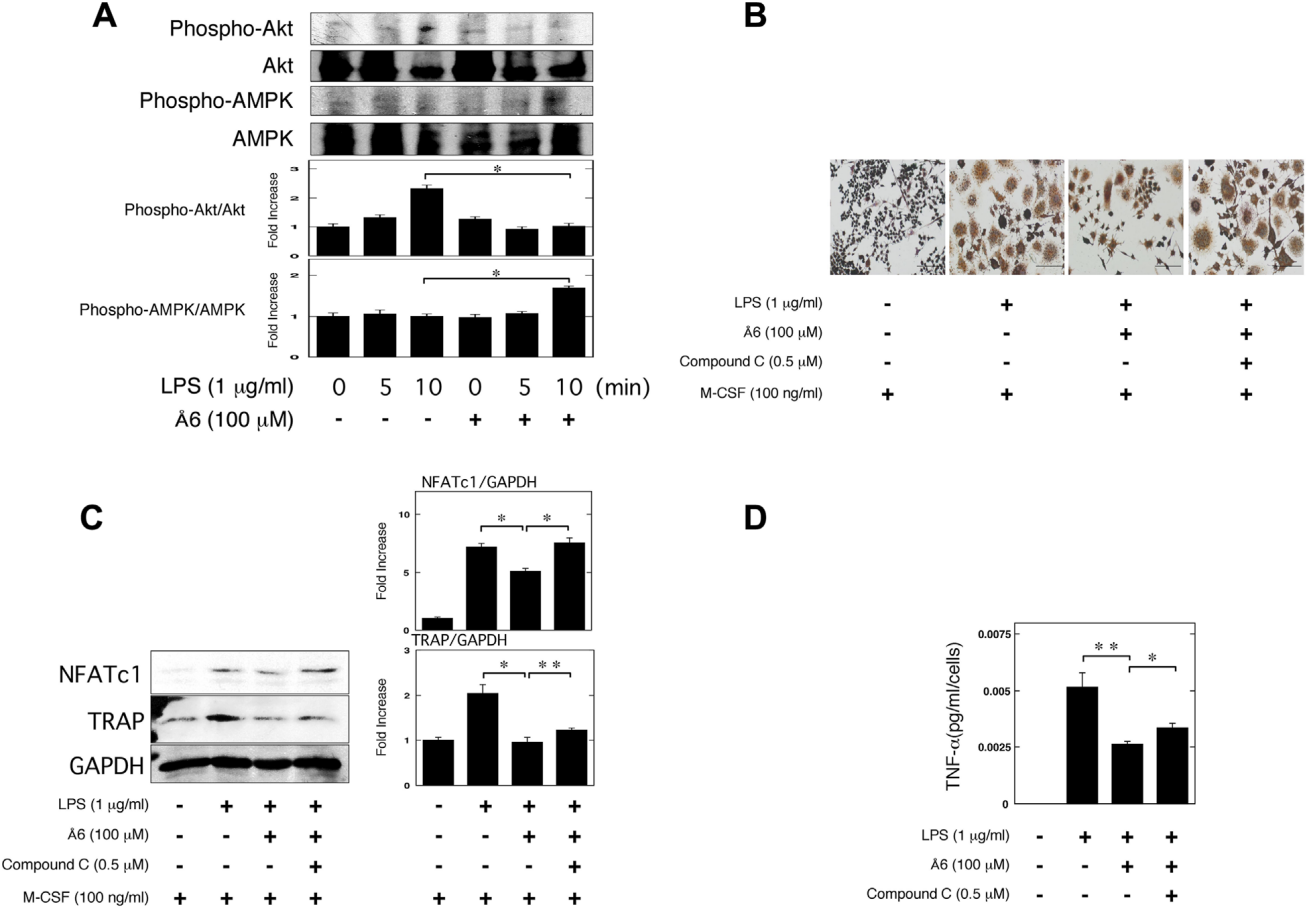
Å6 inhibited the Akt pathway, but activated the AMPK pathway. (A) RAW264.7 cells were pretreated with 100 μM Å6 for 30 min and then stimulated with 1 μg/ml LPS for the indicated periods. Phospho‐Akt, Akt, phospho‐AMPK, and AMPK were examined by a Western blot analysis. The histogram on the bottom panel shows quantitative representations of phospho‐Akt or phospho‐AMPK obtained from densitometry analysis after normalization to the levels of Akt or AMPK expression, respectively (*n* = 3). (B and C) RAW264.7 cells were cultured for 3 days in the absence or presence of LPS (1 μg/ml), M‐CSF (100 ng/ml), Å6 (100 μM), or compound C (0.5 μM) as indicated. (B) TRAP‐staining was performed to detect OC differentiation. Scale bar = 100 μm. (C) The expression of NFATc1 and TRAP in RAW264.7 cells was examined by a Western blot analysis. The histogram on the right panel shows quantitative representations of NFATc1 or TRAP obtained from densitometry analysis after normalization to the levels of GAPDH expression (*n* = 3). (D) RAW264.7 cells were cultured for 24 h in the absence or presence of LPS (1 μg/ml), Å6 (100 μM), or compound C (0.5 μM) as indicated. The TNF‐α content in the conditioned media of RAW264.7 cells was determined by using ELISA as described in Materials and Methods (*n* = 3). The data represent the mean ± SEM. *p < 0.01, **p < 0.05.

It has been reported that the Akt pathway induces OC differentiation 28, 29. Conversely, adenosine monophosphate‐activated protein kinase (AMPK) acts as a negative regulator during OC differentiation 30. Therefore, we examined whether or not Å6 is associated with the Akt and AMPK pathways in RAW264.7 cells. We showed that Å6 attenuated the Akt phosphorylation, but promoted the AMPK phosphorylation (Fig. 5A). Next, we examined that the effects of AMPK inhibitor, compound C 31 in the Å6‐attenuated the OC differentiation induced by LPS. Compound C inhibited the Å6‐attenuated OC differentiation of RAW264.7 cells induced by LPS (Fig. 5B). Compound C also inhibited the Å6‐attenuated the expression of OC markers, NFATc1 and TRAP induced by LPS (Fig. 5C). Furthermore, compound C inhibited the Å6‐attenuated the TNF‐α production induced by LPS (Fig. 5D).

### No effects of Å6 on the LPS‐induced inflammatory OC differentiation in the uPA or uPAR knockdown conditions

**Figure 6:**
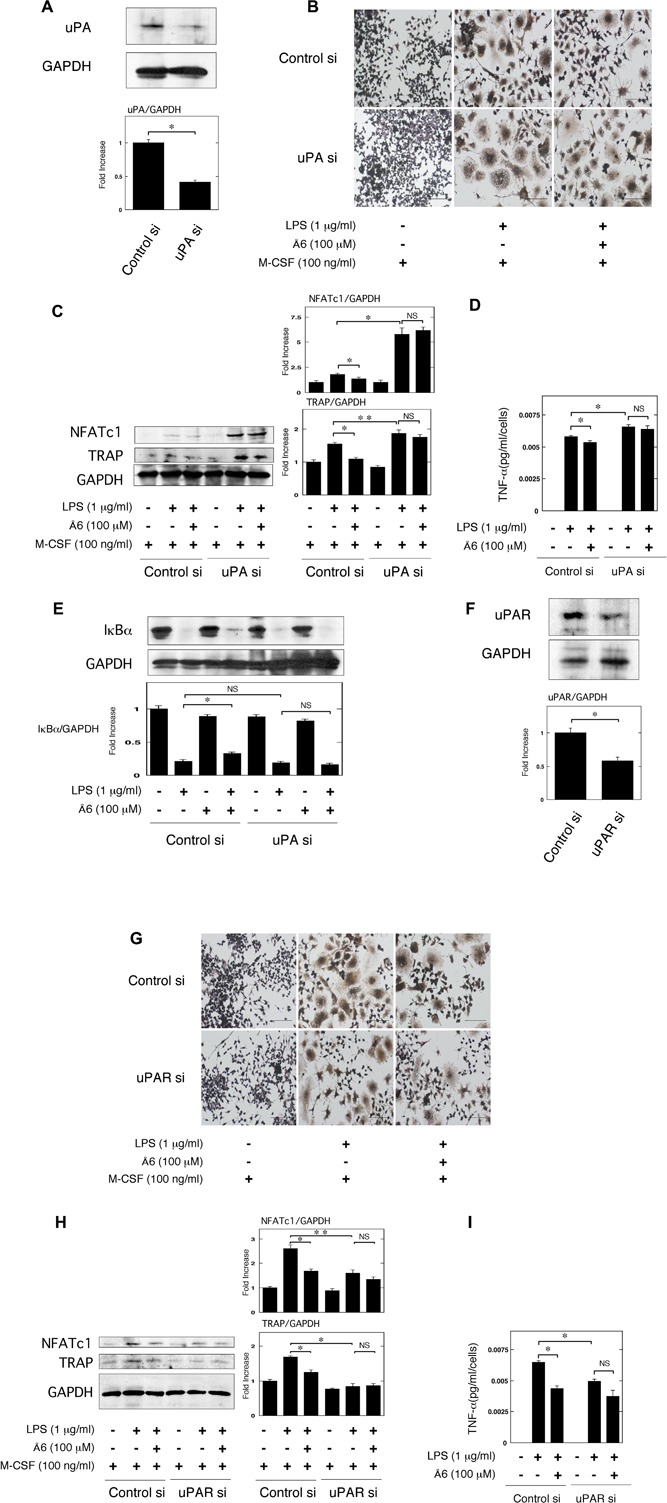
No effects of Å6 on the LPS‐induced inflammatory OC differentiation in the uPA or uPAR knockdown conditions. (A) Status of uPA expression in RAW264.7 cells transfected with control or uPA siRNA was examined by a Western blot analysis. The histogram on the bottom panel shows quantitative representations of uPA obtained from densitometry analysis after normalization to the levels of GAPDH expression (*n* = 3). (B and C) Firstly, either control or uPA siRNA RAW264.7 cells were cultured for 3 days in the absence or presence of LPS (1 μg/ml), M‐CSF (100 ng/ml), or Å6 (100 μM) as indicated. (B) TRAP‐staining was performed to detect OC differentiation. Scale bar = 100 μM. (C) The expression of TRAP and NFATc1 in RAW264.7 cells with control or uPA siRNA was examined by a Western blot analysis. The histogram on the right panel shows quantitative representations of NFATc1 or TRAP obtained from densitometry analysis after normalization to the levels of GAPDH expression (*n* = 3). (D) RAW264.7 cells were cultured with either control or uPA siRNA for 24 h in the absence or presence of LPS (1 μg/ml) or Å6 (100 μM) as indicated. The TNF‐α content in the conditioned media of RAW264.7 cells transfected with control or uPA siRNA was determined by using ELISA as described in Materials and Methods (*n* = 3). (E) RAW264.7 cells were pretreated with Å6 (100 μM) for 30 min and then stimulated with LPS (1 μg/ml) for 15 min. Degradation of IκBα was evaluated by a Western blot analysis. The histogram on the bottom panel shows quantitative representations of IκBα obtained from densitometry analysis after normalization to the levels of GAPDH expression (*n* = 3). (F) Status of uPAR expression in RAW264.7 cells transfected with control or uPAR siRNA was examined by a Western blot analysis. The histogram on the bottom panel shows quantitative representations of uPAR obtained from densitometry analysis after normalization to the levels of GAPDH expression (*n* = 3). (H and I) Firstly, either control or uPAR siRNA RAW264.7 cells were cultured for 3 days in the absence or presence of LPS (1 μg/ml), M‐CSF (100 ng/ml), or Å6 (100 μM) as indicated. (G) TRAP‐staining was performed to detect OC differentiation. Scale bar = 100 μM. (H) The expression of TRAP and NFATc1 in RAW264.7 cells with control or uPAR siRNA was examined by a Western blot analysis. The histogram on the right panel shows quantitative representations of NFATc1 or TRAP obtained from densitometry analysis after normalization to the levels of GAPDH expression (*n* = 3). (I) RAW264.7 cells were cultured with either control or uPAR siRNA for 24 h in the absence or presence of LPS (1 μg/ml) or Å6 (100 μM) as indicated. The TNF‐α content in the conditioned media of RAW264.7 cells transfected with control or uPAR siRNA was determined by using ELISA as described in Materials and Methods (*n* = 3). *p < 0.01, **p < 0.05, NS, not significant.

In previous study, we demonstrated that uPA knockdown promoted the LPS‐induced OC differentiation 11. Conversely, uPAR knockdown attenuated them 12. Here, we examined that the effects of Å6 on the LPS‐induced OC differentiation in the uPA or uPAR knockdown condition. First, we confirmed that uPA siRNA suppressed the uPA expression but control siRNA did not at protein level in the RAW264.7 cells (Fig. 6A). Å6 inhibited the LPS‐induced OC differentiation, TNF‐α production, and IκBα degradation in the control condition, whereas Å6 had no effects on them in the uPA knockdown condition (Fig. 6B–E). Next, we examined that the effects of Å6 on the LPS‐induced OC differentiation in the uPAR knockdown condition. We confirmed that uPAR siRNA suppressed the uPAR expression but control siRNA did not at protein level in the RAW264.7 cells (Fig. 6F). Å6 inhibited the LPS‐induced OC differentiation and TNF‐α production in the control condition, whereas Å6 had no effects on them in the uPAR knockdown condition (Fig. 6G–I).

## Discussion

We herein showed the uPA‐derived peptide, Å6 attenuated LPS‐induced inflammatory osteoclastogensis and bone loss in mice (Fig. 1). We also showed that Å6 attenuated the LPS‐promoted OC differenatiation (Fig. 2). Furthermore, Å6 attenuated the production of TNF‐α (Fig. 3), which is positively associated with the LPS‐induced OC differentiation 26, 27. These data strongly suggest that Å6 is involved in the suppression of LPS‐induced inflammatory osteoclastogenesis and bone loss by attenuation of secretion of the inflammatory cytokine from macrophages that homed into the LPS‐induced inflammatory tissue.

We previously demonstrated that uPA‐activated AMPK attenuated the LPS‐induced NF‐κB activation, and is involved in the suppression of LPS‐induced inflammatory osteoclastogenesis and bone loss 11. Conversely, uPAR‐activated Akt is involved in the promotion of LPS‐induced inflammatory osteoclastogenesis and bone loss 12. We herein showed that Å6 activated the AMPK signaling (Fig. 5A), and attenuated the LPS‐induced NF‐κB activation (Fig. 4). We also showed that the inhibition of AMPK inhibited the Å6‐attenuated OC differentiation induced by LPS (Fig. 5B and C). These data suggest that the Å6‐attenuated inflammatory osteoclastogenesis is associated with the AMPK activation. On the other hand, Å6 inhibited the Akt signaling (Fig. 5A). The activation of Akt is known to inhibit the AMPK pathway 32. It has been reported that uPA promotes the Akt activation 33, and the downregulation of uPA inhibits the Akt signaling 34, 35. In addition, we previously demonstrated that uPAR deficiency or uPAR blocking attenuated the Akt pathway 8, 12, 36. Here, we demonstrated that Å6 inhibited the LPS‐induced OC differentiation, and TNF‐α production in the control condition, whereas Å6 had no effects on them in the uPA or uPAR knockdown conditions (Fig. 6). These results suggested that Å6 functioned as an attenuator of LPS‐induced osteoclastogenesis through the disruption of interaction between uPA and uPAR.

**Figure 7:**
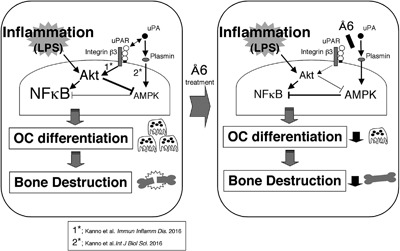
The proposed mechanism of the Å6‐attenuated inflammatory osteoclastogenesis and bone destruction induced by LPS. LPS induced NF‐κB activation, resulting in inflammatory osteoclastogenesis and bone destruction. On the other hand, uPAR and uPA‐activated plasmin activated the Akt and AMPK pathways, respectively. In addition, the uPAR‐induced Akt activation inhibited AMPK pathway. Å6 attenuated the uPAR‐activeted Akt pathway, possibly resulting in the upregulaton of AMPK activation. The resultant suppression of NF‐κB activity inhibited inflammatory osteoclastogenesis and bone destruction.

Thus, LPS induced NF‐κB activation, resulting in inflammatory osteoclastogenesis and bone destruction. On the other hand, uPAR and uPA‐activated plasmin activated the Akt and AMPK pathways, respectively. In addition, the uPAR‐induced Akt activation inhibited AMPK pathway. Å6 attenuated the uPAR‐activeted Akt pathway, possibly resulting in the upregulaton of AMPK activation. The resultant suppression of NF‐κB activity inhibited inflammatory osteoclastogenesis and bone destruction (Fig. 7).

Å6 has multiple functions, such as inhibition of angiogenesis, cell growth, cell migration, cell invasion 13. Angiogenesis, cell growth, cell migration, and cell invasion are known to link to inflammatory bone destruction 37, 38. These functions of Å6 may also affect the suppression of inflammatory osteoclastogenesis and bone loss. Furthermore, it has been reported that Å6 was no toxicity in Phase 1 and 2 clinical trials 18, 19, 20, 21, Å6 might be available for the therapy of inflammatory bone destruction.

In conclusion, uPA‐derived peptide, Å6 is involved in the suppression of LPS‐promoted inflammatory osteoclastogenesis and the resultant bone loss. These findings provide a basis for therapeutic strategies for the inflammatory bone disease.

## Authors’ Contributions

YK conceived and designed the experiments. YK, CM, AM, AI were involved in the experiments. YK analyzed the data. YK and AI wrote the manuscript.

## Conflict of Interest

All authors state that they have no conflicts of interest.
